# How are women living with HIV in France coping with their perceived side effects of antiretroviral therapy? Results from the EVE study

**DOI:** 10.1371/journal.pone.0173338

**Published:** 2017-03-06

**Authors:** Guillemette Quatremère, Marguerite Guiguet, Patricia Girardi, Marie-Noëlle Liaud, Coline Mey, Cynthia Benkhoucha, Franck Barbier, Graciela Cattaneo, Anne Simon, Daniela Rojas Castro

**Affiliations:** 1 AIDES, Pantin, France; 2 Sorbonne Universités, UPMC Univ Paris 06, INSERM, IPLESP UMRS 1136, Paris, France; 3 CIDAG, Department of Internal Medicine, AP-HP Hôpital Pitié-Salpétrière, Paris, France; 4 Social Psychology Research Group (GRePS), University of Lyon 2, Lyon, France; 5 SESSTIM, Inserm U912, Marseille, France; British Columbia Centre for Excellence in HIV/AIDS, CANADA

## Abstract

**Objective:**

Side effects of antiretroviral therapy (ART) can have a negative impact on health-related quality of life threatening long-term retention in HIV care and adherence to ART. The aim of the French community-based survey EVE was to document personal experiences with side effects, the related physician-patient communication, and solutions found to deal with them.

**Design:**

Cross-sectional study of women between September 2013 to September 2014

**Methods:**

An anonymous online questionnaire included the HIV Symptom Distress Module, which explores 20 symptoms.

**Results:**

In all, 301 women on ART participated in the study (median age: 49 years; median duration of ART: 14 years). They reported having experienced a median of 12 symptoms (Q1-Q3: 9–15) during the previous 12 months. Overall, 56% of them reported having found at least a partial solution to dealing with their symptoms. Women reporting financial difficulties were twice less likely to have found solutions to coping with their side effects (AOR: 0.5; 95% CI: 0.3–0.8). Feeling supported by the health-care provider (AOR: 2.1; 95% CI: 1.1–3.9) and being in contact with HIV/AIDS organisations (AOR: 1.9; 95% CI: 1.2–3.2) were positively associated with coping. Seventeen percent reported having modified their ART regimen to improve tolerance, with only 2 in 3 informing their physician afterwards. Reporting financial difficulties and living with more bothersome symptoms increased the risk of ART regimen modification without health-care provider consultation.

**Conclusion:**

The EVE study has called attention to the large number of side effects experienced by WLWHIV, only half of whom have found self-care strategies to manage their symptoms. Modification of ART regimen by the women themselves was not uncommon.

## Introduction

Despite improvement of antiretroviral medication tolerance, side effects of antiretroviral medication therapy (ART) are still frequent and longer time with diagnosed HIV infection has been related to a higher prevalence of perceived distressing symptoms and lower quality of life [1]. Indeed, side effects negatively impact the health-related quality of life of people living with a chronic disease such as HIV [2]. Additionally, side effects have been consistently reported to influence adherence to ART [3–6]. Yet, lifelong treatment with uninterrupted suppressed HIV viral load is necessary to obtain a long-term immunological response, to reduce morbidity and mortality, and to achieve the UNAIDS 90-90-90 targets to help end the HIV/AIDS epidemic [7].

Women living with HIV (WLWHIV), who represent 48% of people living with HIV in the world in 2016 [8], seem to be more affected by side effects of ART. No clear sex difference in efficacy of ART has been observed, but lower adherence, and a higher number of adverse reactions in WLWHIV compared to men has been reported [9,10]. Sex-related differences in pharmacokinetics could explain variation in toxicity that has been observed between women and men [11]. Gender-specificities have been observed in treatment-related side effects with all classes of antiretroviral drugs. In particular, ART discontinuation for neurologic, dermatologic, or constitutional toxicities has been more frequently reported among women [12]. Another example is type of fat redistribution that varied according to sex [13]. Even with newer drugs, women appeared to be more at risk of neuropsychiatric adverse events than men [14]. However, WLWHIV are still under-represented in clinical trials of ART preventing a global view of ART tolerance in women [15]. The first clinical trial done exclusively among women showed a difference in ART discontinuation due to adverse events according to ART regimens which has not been found in previous clinical trials [16].

Differences are also observed among women regarding their HIV infection and ART. Interestingly, the WAVES trial [16] which enrolled women from 13 countries reported regional differences in efficacy, drug adherence or discontinuation of study drugs, underlying the role of socioeconomic factors. Social, economic, and behavioral factors have been associated with adherence, especially among vulnerable groups such as transgender women [17], or women who use drugs [18]. More generally, factors such as educational level have been shown to modify virological response as well as survival among PLWHIV receiving ART with the largest proportion of women in lower educational strata [19].

Side effects can have consequences on trust in the health-care provider [20], which is important given that the quality of the patient-provider relationship has been shown to be a key factor for long-term retention in HIV care [21]. Gender differences in the relationships with HIV care providers can be indirect, for instance in the case of depression. The patient-provider relationship has been found to be impaired by depressive symptoms and it has been reported that women had higher depression scores than men [22]. The Women Living Positive survey highlighted the communication barriers with their health-care providers, experienced by women receiving ART, with a majority who had never discussed gender-issues. Meanwhile, many thought that HIV-infection and treatment affect women differently than men [23].

In 2011, during the “Positive women in action!” workshop, organised by WLWHIV at AIDES, a French community-based organization (CBO) involved in the fight against HIV/AIDS, the participants had an opportunity to talk about their difficulties dealing with daily side effects and the communication barriers with their health-care providers. In particular, they shared the feeling of being constantly referred for psychosocial issues rather than for objective investigations into their experienced side effects. They decided to conduct, in partnership with researchers and practitioners, a study of side effects experienced by WLWHIV. This article presents the results of the EVE (EVEnts) study, which was designed with the objectives of quantifying the extent and severity of common side effects and documenting dialogue with health-care providers and coping methods.

## Methods

This cross-sectional study was designed in accordance with the principles of community-based research [24]. WLWHIV were involved in the initiation of the study and its design, and they remained involved throughout, the dissemination of the survey, the analysis and the released of results, in partnership with scientists and practitioners.

### Data collection

A self-administered online questionnaire was freely accessible at a community-based website for one year (September 2013 to September 2014), and a paper version was available at AIDES’s offices. The EVE survey was intended for women living with HIV including trans (male to female or female to male). The two exclusion criteria were to be a man and to be not infected with HIV. The survey was promoted on social networks, on AIDES’s website and during the CBO’s activities. Respondents completed an anonymous questionnaire with no identifying details on a voluntary basis. The IP addresses of the respondents who completed the questionnaire online were not collected.

The EVE survey was an observational study with data anonymously collected. No data made possible the identification of the respondents. The self-administered online questionnaire was accessible at a community-based website (aides.org, CNIL N° 755883). However, no specific data protection office approval was looked for the EVE survey specifically. According to European legislation: "The principles of data protection should therefore not apply to anonymous information, namely information which does not relate to an identified or identifiable natural person […]. Regulation does not therefore concern the processing of such anonymous information, including for statistical or research purposes." (Regulation (EU) 2016/679 Of The European Parliament And Of The Council of 27 April 2016 on the protection of natural persons with regard to the processing of personal data and on the free movement of such data, and repealing Directive 95/46/EC).

A few sociodemographic characteristics (age, country of birth, level of education, employment status and financial difficulties) and health data (body mass index [BMI], HBV or HCV co-infections, comorbidities, and duration of HIV infection and ART) were recorded. Side effects were assessed using a 20-item scale, the HIV Symptom Distress Module (SDM) [25]. The time period for reporting symptoms was extended for 12 months instead of 4 weeks, the period in the original module, which was designed mainly for clinical trials. For each symptom, questions were asked about the degree of bother for three areas: work-related activities, social and family life, and intimacy and sexual life. The degree of bother was rated as follows: “do not have symptom”, “have symptom, but no bother”, “have symptom, little bother”, “have symptom, bother”, “have symptom, bothers me a lot”. A given symptom was classified as “bothersome” if the respondent selected either of the last two degrees of bother in at least one area. Each symptom was also investigated with regard to the related physician-patient dialogue. Finally, the method for dealing with side effects was investigated. Respondents were asked to report all the solutions their health-care provider had given them and, more generally, all the solutions they had tried. One specific question investigated whether they had modified their ART regimen, such as by decreasing the dosage or increasing the dosing intervals, to help alleviate their side effects.

According to the French legislation, the EVE survey was an observational study with data anonymously collected. This survey was not under any ethic committee referral.

### Statistical methods

Descriptive statistics are shown as medians (first quartile-third quartile [Q1-Q3]) or frequencies (%), with the comparisons based on the Kruskal-Wallis test for continuous variables and on χ^2^ tests or the Fisher exact test for categorical variables. Two sets of analyses were performed to study 1) factors associated with having found a solution to deal with side effects; and 2) factors associated with a change to the ART regimen without consulting the health-care provider. A separate multivariable logistic model was used for each of the two outcome variables (solution and change in ART regimen). A backward stepwise selection was used to select the final multivariable model. All the variables possibly associated (p<0.10) with the outcome of interest in the univariable analyses were first included. Then variables with the highest Type III p-value were iteratively removed until no variable was left. The final model was the model with the lowest Akaike Information Criterion (AIC). The results were presented as adjusted odds ratios (AORs) and 95% confidence intervals (CIs). The statistical analyses were performed with SAS v9.3 software (SAS Institute Inc.).

## Results

In all, 315 women took part in the study. The analyses were limited to the 301 (96%) WLWHIV who were on ART (Table 1). The median age was 49 years, and the median duration since HIV diagnosis was 18 years. It should be noted that 147 (49%) women reported financial difficulties (“I can’t manage to live without being in debt.” or “Financially, things are difficult”). More than half of the women had personally been in contact with HIV/AIDS organisations. The median duration of ART was 14 years, with 46 (15%) having been on ART for more than 20 years. The CD4 count was above 500 cells/mm^3^ in 55% of the women, and the HIV viral load was controlled in 86% of them.

**Table 1:** Sociodemographic and clinical characteristics of the 301 women living with HIV who participated in the EVE study (2014).

		Median (Q1-Q3) or N (%)
Age, years		49 (42, 54)
Foreign-born		86 (28.8)
High level of educational attainment *		117 (38.9)
Living in a couple		104 (35.1)
Has children		186 (61.8)
Employment status		
	Employed	117 (38.9)
	Unemployed	52 (17.3)
	Disability	71 (23.6)
	Retired, other	61 (20.3)
Financial difficulties		147 (49.8)
Contact with HIV/AIDS organisations		174 (59.0)
Body mass index (BMI), kg/m^2^		22.7 (20.0, 27.3)
Hepatitis B co-infection		11 (3.8)
Hepatitis C co-infection		46 (15.6)
Chronic disease		71 (23.6)
Time since HIV diagnosis, years		18 (9, 24)
Time since ART initiation, years		14 (7, 18)
CD4 cell count/mm^3^		
	<350	52 (18.6)
	350–500	74 (26.4)
	>500	154 (55.0)
HIV RNA <50 copies/mL		259 (86.1)

* College graduate

Missing data: age (n = 4); living in a couple (n = 5); financial difficulties (n = 6); body mass index (n = 15); contact with HIV/AIDS organisations (n = 2); years since HIV diagnosis (n = 1); years since ART initiation (n = 11).

**Fig 1:**
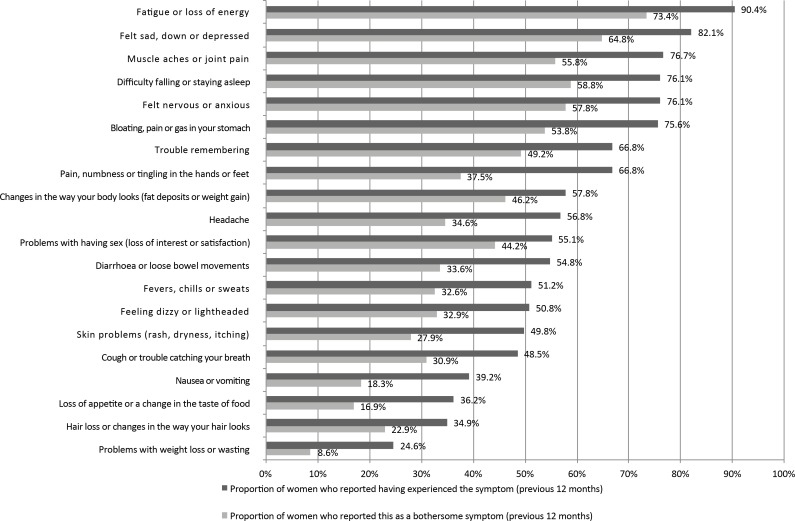
Proportion of women who reported having experienced the symptom during the previous 12 months and the proportion of women who reported having found the symptom bothersome: the EVE study (France, 2014).

All the respondents reported having had at least one symptom during the previous year, and, as shown in Fig 1, the symptoms reported most frequently were also those that had a greater negative impact on the WLWHIV’s daily life. Fatigue or loss of energy was the main symptom. Sleep disturbances, depression and anxiety were reported by the majority of the women. Muscle aches or joint pain was bothersome in 56% of the women. Gastrointestinal symptoms were still common, with 101 (34%) suffering from diarrhoea and 162 (54%) from bloating. Body changes and sexual issues were problems for 46% and 44% of the women, respectively. The median number of symptoms experienced was 12 (Q1-Q3: 9–15), and the median number of bothersome symptoms was 8 (Q1-Q3: 4–11).

As regards fatigue and body modification, more than 80% of the women who reported these symptoms indicated that they had talked to their doctors about them. Similarly, a majority of the women with depression, sleep disturbances, muscle aches or gastrointestinal symptoms talked to their doctor about these issues. On the subject of sexual problems, only 29% had talked about them in a medical care setting. For fatigue, body modification and sexual problems, only half of the women who had talked about them felt that their doctor had actually listened to them. The dialogue was slightly better for depression (70%) and physical symptoms, such as peripheral neuropathy (64%) and diarrhoea (66%). Most of the respondents (80%) indicated that a health-care provider had proposed a solution for at least one symptom. Among the solutions provided by health professionals were a referral to another specialist (45%), a drug prescription (45%), a change of antiretroviral drug (24%), and ARV titration (8%).

Overall, 56% of the women reported having found a solution to deal with their symptoms, at least partly (Table 2). In multivariable analysis, the women who reported financial difficulties were twice less likely to have found solutions to cope with their side effects (AOR, 0.5; 95% CI: 0.3–0.8), while feeling supported by the health-care provider (AOR: 2.1; 95% CI: 1.1–3.9) and being in contact with HIV/AIDS organisations (AOR: 1.9; 95% CI: 1.2–3.2) were positively associated with coping. The likelihood of coping decreased as the number of bothersome symptoms increased (AOR: 0.9 per additional symptom; 95% CI: 0.9–1.0). The most frequent nonexclusive solutions used by the women themselves were physical activity (28%), manual therapy (25%), and nutritional supplements (26%). Phytotherapy and homeopathy were utilized frequently as well (24%), while the therapeutic use of cannabis was less common (6%). Help with daily and domestic activities (9%) and work-time arrangements (8%) were also mentioned. To relieve side effects, 51 (17%) reported having made a change to their ART regimen on their own, such as decreasing dosage or increasing the dosing intervals, without prior notice to their health-care provider, and only two-thirds of these women informed their physician afterwards. The sociodemographic variables education and financial difficulties were associated with a change to the ART regimen without prior discussion (Table 3). Women living with hepatitis co-infection and those who were underweight or overweight were more likely to have modified their ART regimen without consulting their health-care provider. The women who had decided to modify their ART reported a higher number of bothersome symptoms than those who did not make such a change (median: 10 vs. 8; *p* = 0.006). In multivariable analysis, reporting financial difficulties (AOR: 2.0; 95% CI: 1.0–4.0) and living with more bothersome symptoms (AOR: 1.1 per additional symptom; 95% CI: 1.0–1.2) increased the risk of ART regimen modification without prior health-care provider consultation. Women who were underweight were more likely to change ART regimen (AOR: 2.6; 95%CI: 1.0–6.6) while being overweight was not associated with ART regimen modification (AOR: 1.7; 95%CI: 0.8–3.4).

**Table 2:** Differences in characteristics between women who reported having found a solution to relieving their side effects and those who did not: the EVE study (2014).

		Solution found N (Col %)	No solution N (Col %)	p
All		170 (100.0)	131 (100.0)	
Age ≥ 50 years		81 (48.2)	51 (39.5)	0.16
Born in France		124 (72.9)	88 (67.2)	0.31
High level of educational attainment		76 (44.7)	41 (31.3)	0.02
Living in a couple		57 (34.1)	47 (36.4)	0.71
Has children		107 (62.9)	79 (60.3)	0.72
Employment status				0.24
	Employed	73 (42.9)	44 (33.6)	
	Unemployed	24 (14.1)	28 (21.4)	
	Disability	38 (22.3)	33 (25.2)	
	Retired, other	35 (20.6)	26 (19.9)	
Financial difficulties		70 (41.9)	77 (60.2)	0.002
Contact with HIV/AIDS organisations		112 (65.9)	64 (49.6)	0.006
BMI, kg/m^2^				0.38
	<18.5	18 (11.0)	15 (12.2)	
	18.5–24.9	89 (54.6)	57 (46.3)	
	≥25	56 (34.4)	51 (41.5)	
Hepatitis B or C co-infection		32 (18.8)	21 (16.0)	0.55
Chronic disease		43 (25.3)	28 (21.4)	0.49
Years since HIV diagnosis (median[Q1-Q3])		18 (8–24)	18 (11–24)	0.50
Years since ART initiation (median [Q1-Q3])		13 (6–18)	15 (8–18)	0.24
Solution proposed by health-care provider		144 (85.7)	89 (71.8)	0.005
Number of bothersome symptoms (median [Q1-Q3])		8 (4–11)	9 (4–12)	0.06

Missing data: age (n = 4); living in a couple (n = 5); financial difficulties (n = 6); body mass index (n = 15); contact with HIV/AIDS organisations (n = 2); years since HIV diagnosis (n = 1); years since ART initiation (n = 11); solution proposed by health-care provider (n = 9).

**Table 3:** Differences in characteristics between women who reported having modified their ART regimen by decreasing the dosage or increasing the dosing intervals without prior consulting their health-care provider and those who did not: the EVE study (2014).

		Modification in ART regimen without prior consultation N (Col %)	No modification to ART regimen or modification after consultation N (Col %)	p
All		51 (100.0)	250 (100.0)	
Age ≥ 50 years		20 (40.0)	112 (45.3)	0.53
Born in France		35 (68.8)	177 (70.8)	0.74
High level of educational attainment		13 (25.5)	104 (41.6)	0.04
Living in a couple		13 (26.0)	91 (37.0)	0.15
Has children		37 (72.5)	149 (59.6)	0.11
Employment status				0.20
	Employed	15 (29.4)	102 (40.8)	
	Unemployed	7 (13.7)	45 (18.0)	
	Disability	17 (33.3)	54 (21.6)	
	Retired, other	12 (23.5)	49 (19.6)	
Financial difficulties		35 (68.6)	112 (45.9)	0.003
Contact with HIV/AIDS organisations		28 (54.9)	148 (59.7)	0.53
BMI, kg/m2				0.04
	<18.5	9 (18.0)	24 (10.2)	
	18.5–24.9	18 (36.0)	128 (54.2)	
	≥25	23 (46.0)	84 (35.6)	
Hepatitis B or C co-infection		14 (27.5)	39 (15.6)	0.07
Chronic disease		15 (29.4)	56 (22.4)	0.28
Years since HIV diagnosis (median[Q1-Q3])		20 (8–26)	17 (9–24)	0.12
Years since ART initiation (median[Q1-Q3])		15 (8–18)	13 (6–18)	0.27
Solution proposed by health-care provider		41 (80.4)	192 (79.7)	1.0
Number of bothersome symptoms (median [Q1-Q3])		10 (7–12)	8 (4–11)	0.006

Missing data: age (n = 4); living in a couple (n = 5); financial difficulties (n = 6); body mass index (n = 15); contact with HIV/AIDS organisations (n = 2); years since HIV diagnosis (n = 1); years since ART initiation (n = 11); solution proposed by health-care provider (n = 9).

## Discussion

The results of the EVE study document the severity of common side effects experienced by women living with HIV and the difficulties encountered in the physician-patient relationship regarding them. EVE respondents reported having experienced a median of 12 symptoms during the previous year. Not all the women had talked about their symptoms with their health-care provider, and those who did widely reported the feeling of not having been listened to. Physical activity and manual therapy were frequently reported as being helpful, as were herbals, while 44% of the women had not found any solution to relieving their symptoms. Furthermore, 17% reported having modified their ART regimen to improve tolerance by decreasing the dosage or increasing the dosing intervals, without prior discussion with their health-care provider. Reporting financial difficulties decreased the likelihood of coping and was associated with changes to the ART regimen without prior discussion.

The strength of this study was the involvement of WLWHIV throughout the entire process. Confidence in the CBO AIDES facilitated the participation of women who had been living with HIV for a prolonged period of time, with half of the respondents experiencing social vulnerability. However, our study has some limitations. The women who answered the EVE survey were not a representative sample of WLWHIV. Questionnaires were in French only. Survey was online and partially completed online questionnaires were not retained in the database. Online survey missed women with no internet access. To overcome selective recruitment, paper questionnaires were available in local AIDES offices with translation help. Data entry for 53 (17%) paper questionnaires was performed by AIDES volunteers but, unfortunately, it was not possible to distinguish completion modes in the analyses. Furthermore, we modified the recall period from 4 weeks to 12 months in order to capture long-term side effects that negatively correlated with quality of life. This could explain why all the women in the EVE survey reported having experienced at least one symptom and why there was a high number of symptoms. However, in a recent and representative national survey of people living with HIV followed at hospitals in France [26] that included a 22-item symptom questionnaire derived from the HIV Symptom Distress Scale [25], 96% of those on ART reported at least one symptom during the previous 4 weeks, with a median number of symptoms of 9 (VESPA2, personal communication). Another limitation was the lack of data concerning ART regimen. In a context of a large availability of antiretroviral drugs, modification of antiretroviral therapy is frequent in France, with a median number of seven antiretroviral regimens reported in a recent study, including individuals who have been on ART for a median duration of 12 years [27]. In our cross-sectional survey with no access to medical record, a testing phase showed the difficulty to retrospectively collect longitudinal information on antiretroviral therapy and onset of side effects. In this self-administering questionnaire, no data was collected about smoking, alcohol consumption or drug use though these behavioral factors could modify the experience of side effects. Lastly, it was not possible to investigate whether some means of coping were more helpful for specific symptoms because of the large number and diversity of the symptoms reported by each woman.

The women in the EVE study reported many nontherapeutic ways of dealing with side effects. One-quarter of them indicated that they had engaged in physical activity to relieve some of their side effects. Physical activity has been recognized as being beneficial for body morphology [28] and is associated with a better quality of life [29]. This effective, nonmedical solution should be encouraged by health-care providers, and an interventional trial of physical activity should explore its impact on side effects. Previous studies have documented herbal remedy and supplement use in people on ART [30,31] for improving their quality of life [32]. These complementary alternative medicines could be challenging because of potential drug interactions with ARVs [33], but disclosure to health-care providers has been reported to be far from systematic [31]. The concomitant administration of complementary treatments should be reviewed systematically, as their use has been reported to be widely shared among all groups, independently of personal characteristics [34].

Despite the declining use of the first and more toxic antiretroviral drugs, tolerability is still a major issue, and side effects have been reported as a common reason for missing ART [6]. In the EVE study, some women had decided to decrease the dosage or increase the ARV dosing intervals without consulting their health-care provider, thereby running the risk of decreased effectiveness. In fact, a lower rate of viral suppression was observed among these women than among those who did not do this (75% vs. 88%; *p* = 0.01). Decreasing ARV intake is not necessarily itself. Recent clinical trials have shown that certain selected ART regimens can be effective with a weekends-off schedule and entail less severe side effects [35], or that switching to a lower dose of Atazanavir was effective with less toxicity than the standard dose among Thai PLWHIV with mean bodyweight of 59 kg which is closer to women’s bodyweight [36]. In addition, it has been long advocated that health-related quality of life be considered in addition to efficacy when choosing between the many possible combinations of antiretroviral therapies [37]. Communication between women and their health-care providers is even more essential here to prevent the negative consequences of making a change to their ART regimen without medical supervision.

In the EVE study, having financial difficulties decreased the likelihood of coping with symptoms and increased the risk of ART regimen modification without health-care provider consultation. Self-reported financial difficulties could apply to different situations, but the relationship between socioeconomic factors and worse health outcomes in HIV care has been observed in different contexts. In countries where not having private health insurance could indicate socioeconomic difficulties, this factor has been associated with ART discontinuation and loss to follow-up [38]. Other socioeconomic indicators, such as a lack of stable housing, have also been shown to be associated with lower adherence to HIV therapy [39]. Furthermore, social vulnerability negatively impacts satisfaction with physicians [40] and, as a result, physician-patient communication, which is a factor associated with retention to care [21]. More attention should be given to helping socially vulnerable WLWHIV access symptom- management strategies in order to prevent health-care inequalities.

Community-based HIV organisations can support people living with HIV (PLWHIV) in the management of their disease (empowerment) or help fill gaps in the care provided by the health-care system [41]. In the EVE study, being in contact with an HIV organisation was found to be associated with finding a way of coping. The sharing of experiences and tips between PLWHIV on how to deal with side effects (practical solutions and how to get health-care providers to listen) could explain this finding. The Internet, and specifically community-based support groups on the Internet, can also offer this kind of support [42,43].

## Conclusion

The EVE study has called attention to the large number of side effects experienced by WLWHIV, only half of whom have found self-care strategies to manage their symptoms. Health-care provider support and community support were factors favouring the likelihood of finding an effective way to cope with side effects, while financial difficulties had a negative effect. Community-based participatory research can help by documenting issues of concern for people living with HIV.

## Supporting information

S1 File Database for the symptoms experienced by women in the EVE study. (XLSX)

S2 File Database for the ways of coping declared by the respondents to the EVE study. (XLSX)

S3 File Database with sociodemographic and clinical characteristics of the women who participated in the EVE study. (XLSX)
